# On the (im)possibility of reconstructing plasmids from whole-genome short-read sequencing data

**DOI:** 10.1099/mgen.0.000128

**Published:** 2017-08-18

**Authors:** Sergio Arredondo-Alonso, Rob J. Willems, Willem van Schaik, Anita C. Schürch

**Affiliations:** ^1^​ Department of Medical Microbiology, Universitair Medisch Centrum Utrecht, Utrecht, The Netherlands; ^2^​ Institute of Microbiology and Infection, University of Birmingham, Birmingham, England, UK

**Keywords:** plasmids, mobile genetic elements, DNA sequence analysis, bacterial genomes, replicon benchmarking

## Abstract

To benchmark algorithms for automated plasmid sequence reconstruction from short-read sequencing data, we selected 42 publicly available complete bacterial genome sequences spanning 12 genera, containing 148 plasmids. We predicted plasmids from short-read data with four programs (PlasmidSPAdes, Recycler, cBar and PlasmidFinder) and compared the outcome to the reference sequences. PlasmidSPAdes reconstructs plasmids based on coverage differences in the assembly graph. It reconstructed most of the reference plasmids (recall=0.82), but approximately a quarter of the predicted plasmid contigs were false positives (precision=0.75). PlasmidSPAdes merged 84 % of the predictions from genomes with multiple plasmids into a single bin. Recycler searches the assembly graph for sub-graphs corresponding to circular sequences and correctly predicted small plasmids, but failed with long plasmids (recall=0.12, precision=0.30). cBar, which applies pentamer frequency analysis to detect plasmid-derived contigs, showed a recall and precision of 0.76 and 0.62, respectively. However, cBar categorizes contigs as plasmid-derived and does not bin the different plasmids. PlasmidFinder, which searches for replicons, had the highest precision (1.0), but was restricted by the contents of its database and the contig length obtained from *de novo* assembly (recall=0.36). PlasmidSPAdes and Recycler detected putative small plasmids (<10 kbp), which were also predicted as plasmids by cBar, but were absent in the original assembly. This study shows that it is possible to automatically predict small plasmids. Prediction of large plasmids (>50 kbp) containing repeated sequences remains challenging and limits the high-throughput analysis of plasmids from short-read whole-genome sequencing data.

## Abbreviation

WGS, whole-genome sequencing.

## Data Summary

1. No new sequencing data has been generated in this study. All genomes used in this research are publicly available in the National Center for Biotechnology Information Sequence Read Archive and accession numbers are specified in Table S1 (available with the online Supplementary Material).

2. Scripts, results and detailed analysis of the metrics reported in this manuscript are available through a public GitLab repository (https://gitlab.com/sirarredondo/Plasmid_Assembly.git). This includes assemblies, program results, QUAST output and an extensive report of the R analysis performed.

## Impact Statement

Short-read sequencing of bacterial DNA has become the gold-standard approach to describe bacterial diversity and evolution. However, the assembly of short-read sequencing data will practically always lead to a fragmented genome assembly, complicating the identification of plasmids from assemblies. Recently, a number of tools have been developed to enable the automated prediction and reconstruction of plasmid sequences. Here, we tested these programs on short-read sequencing data sets, by comparing their output with complete genome sequences that were generated using long-read sequencing technologies. None of the tested programs were able to fully and unambiguously predict distinct plasmid sequences. All programs performed best with the prediction of plasmids smaller than 50 kbp. Larger plasmids were only correctly predicted if they were present as a single contig in the assembly. While predictions by PlasmidSPAdes and cBar contained most of the plasmids, they were merged with, or indistinguishable from, other plasmids or chromosomal sequences. PlasmidFinder missed most plasmids, but all its predictions were correct. Without manual steps, or long-read sequencing information, plasmid prediction from short-read sequencing data remains challenging.

## Introduction

A bacterial cell can hold zero, one or multiple plasmids with varying sizes and copy numbers. Traditionally, plasmid sequencing involved methods to purify plasmid DNA, followed by shot-gun sequencing, which frequently necessitated closing of gaps by primer-walking [1]. Plasmid DNA purification is exceedingly difficult if it involves plasmids longer than 50 kbp [1, 2]. Alternatively, plasmid sequences can be assembled from whole-genome-sequencing (WGS) data generated by high-throughput short-read sequencing platforms. However, plasmids often contain repeat sequences that are shared between the different physical DNA units of the genome, which prohibits complete assembly from short-read data. Assembly often results in many fragmented contigs per genome of unclear origin (plasmid or chromosome) [3]. Currently available plasmid prediction programs either aim to determine whether a previously assembled contig is from a plasmid (PlasmidFinder, cBar), or try to reconstruct whole plasmid sequences from the sequencing reads or the assembly graph (Recycler, PlasmidSPAdes, PLACNET) (Table 1).

**Table 1.** Overview of the programs to predict plasmids from short-read sequencing data

**Program**	**Input**	**Paired-end information**	**Coverage**	**k-mer composition**	***De Bruijn* graph**	**Similarity to replicons**	**Similarity to relaxases**	**Similarity to plasmids**	**Web tool**	**Command-line interface**	**Included in the study**
PlasmidFinder [4]	Contigs					✓			✓		✓
cBar [5]	Contigs			✓						✓	✓
Recycler [9]	BAM+assembly graph	✓	✓		✓					✓	✓
PlasmidSPAdes [10]	Reads	✓	✓		✓					✓	✓
PLACNET [6]	BAM/SAM+contigs	✓	✓			✓	✓	✓		✓	

PlasmidFinder is a web-based tool that was developed to detect replicon sequences in assemblies and is optimized for use in enterobacterial genomes [4]. Since two plasmids sharing the same replication mechanism cannot coexist in the long term within the same cell, replicon sequences are used to classify plasmids into different incompatibility groups [4].

cBar was specifically designed to predict plasmid-derived sequences based on differences in k-mer composition [5]. It relies on differences in pentamer frequencies from 881 complete prokaryotic sequences and gives a binary classification of chromosome- or plasmid-derived contig.

PLACNET (plasmid constellation network) reconstructs plasmids from WGS data by integrating three lines of evidence: (i) scaffold linking and coverage information, (ii) presence of replication initiator proteins (Rip) and relaxase proteins (Rel), (iii) similarity of the sequences with a custom database containing non-redundant plasmid sequences from the National Center for Biotechnology Information [6]. Manual pruning in Cytoscape is necessary to obtain disjoint components [7, 8]. Prediction reproducibility rates are thus highly dependent on the expertise of the researcher. As we aimed to test only fully automated methods for plasmid prediction, we excluded PLACNET from the comparison.

More recently, two algorithms that predict plasmids on the basis of the information contained in the de Bruijn graph were published: Recycler [9] and PlasmidSPAdes [10]. Recycler extracts the information from the de Bruijn graph searching for sub-graphs (cycles) corresponding to plasmids. Selection of the cycles is based on the following assumptions: (i) nodes forming a plasmid have a uniform coverage, (ii) a minimal path must be selected between edges because of repetitive sequences, (iii) contigs belonging to the same cycle have concordant paired-end information, and (iv) plasmid cycles exceed a minimum length.

PlasmidSPAdes assumes a highly uniform contig coverage within the chromosome. It calculates the median coverage from the SPAdes assembly graph [11] to estimate a chromosome coverage. PlasmidSPAdes then builds a second assembly graph (referred to as the plasmid graph) only considering contigs with a read contig coverage differing from the chromosome coverage. After repeat resolution using ExSPAnder [12], connected components in the plasmid graph are reported as putative plasmids.

Here, we benchmarked currently available programs starting either from the reads or from assembled contigs. The aim of this study was to determine whether it was possible to obtain complete plasmid sequences in an automated fashion.

## Theory and implementation

At the time the study was conceived (July 2016), we selected all bacterial genomes with complete plasmid sequences and Illumina Miseq or Hiseq paired-end data publicly available. Complete genome sequences and reads were downloaded from GenBank and the National Center for Biotechnology Information Sequence Read Archive, respectively (Table S1). All the strains were previously sequenced by Pacific Biosystems PacBio RS II.

The above criteria resulted in a set of 42 genomes that spanned twelve different genera (Fig. S1). The test data contained 148 plasmid sequences ranging from 1.55 to 338.85 kbp (Fig. S1, Table S1) and 45 chromosomal sequences ranging from 0.93 to 6.26 Mbp. This set included five genomes used in PlasmidSPAdes and Recycler publications to ensure consistency between present and previously published results (Supplementary Results 1 and Table S2).

We used QUAST 4.1 [13] to map the predicted plasmid contigs against (i) each reference plasmid separately or (ii) the reference genome (containing chromosomes and plasmids, Fig. S2). Nucmer alignments were used to assign each of the predicted plasmid contigs to one of the following three categories: ‘plasmid fraction’ (true positive), ‘chromosome fraction’ (false positive) and ‘fraction of novel sequences’ (absent from the reference genomes). A minimum alignment of 500 bp and 95 % nucleotide identity was required to assign a contig to a certain fraction.

We considered the whole contig length to evaluate the performance of the programs using recall and precision.

- Recall was defined as the percentage of the reference plasmid(s) covered by the prediction. On the individual plasmid level, a recall of 1 indicates that the full reference plasmid sequence was present among the predicted plasmids. On the whole genome level, a recall of 1 indicates all the reference plasmids were fully present among the predicted plasmids.
- Precision was defined as the fraction of true positives (plasmid fraction) divided by the sum of true and false positive results (plasmid and chromosome fraction). $Precision=\frac{Plasmidfraction}{\left(Plasmidfraction+Chromosomefraction\right)}$

For each genome (*n*=42), we calculated precision and recall values. To calculate the overall precision and recall of PlasmidSPAdes, Recycler and PlasmidFinder, we excluded the negative control *Burkholderia* *cenocepacia* strain 22E-1 as no false-positive results were obtained. Additionally, the overall precision and recall of PlasmidFinder was calculated filtering out genomes corresponding to Gram-positive bacteria. A detailed explanation of the metrics reported in the paper is available in Supplementary Methods.

### Prediction per single plasmids

**Fig. 1.**
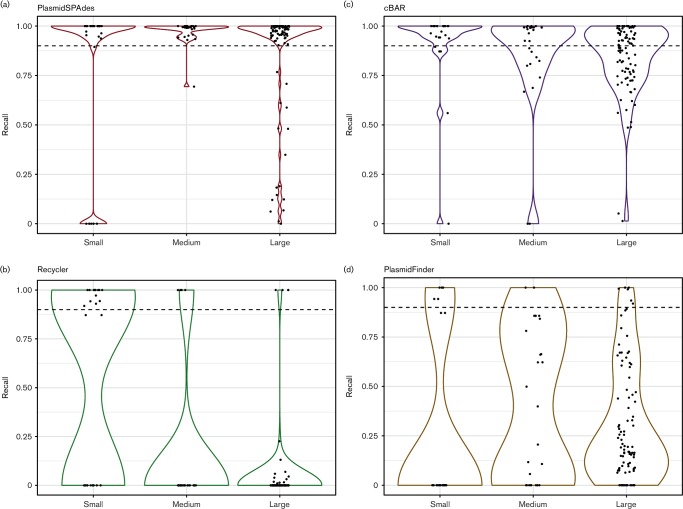
Performance of the programs on a single plasmid level. Recall values of small (less than 10 kbp), medium (from 10 to 50 kpb) and large (greater than 50 kbp) plasmids by PlasmidSPAdes, cBar, Recycler and PlasmidFinder. Recall was calculated by aligning the reference plasmid sequences against the plasmid predictions of each genome and disregarded plasmid binning (if any).

**Fig. 2.**
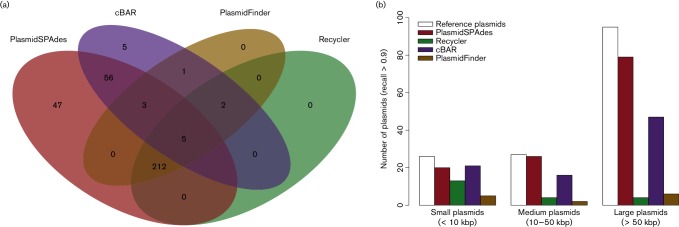
Comparison of program performance on a single plasmid level. (a) A minimum recall value of 0.9 in the program prediction was selected to consider a plasmid as correctly predicted. Venn diagram showing the overlap in prediction between PlasmidSPAdes (red), cBar (purple), PlasmidFinder (orange) and Recycler (green). The intersection of the ellipses showed five plasmids present in all the predictions. (b) Reference plasmids were classified into small (less than 10 kbp), medium (from 10 to 50 kbp) and large (greater than 50 kbp) plasmids depending on their size. The number of reference plasmids correctly predicted (minimum recall value of 0.9) by the programs is represented in the three categories.

We defined a minimum recall value of 0.9 to classify a plasmid as correctly predicted. Out of 148 reference plasmids included in this study, 133 (89.9 %) were correctly predicted by either PlasmidFinder, cBar, Recycler or PlasmidSPAdes (Figs 1 and 2). PlasmidSPAdes correctly predicted 125 plasmids, cBar 84 plasmids, Recycler 21 plasmids and PlasmidFinder 13 plasmids at a recall of 0.9 or more (Figs 1 and 2a, b).

Of all 148 plasmids, 5 plasmids were consistently correctly predicted by all of the programs (Fig. 2a). These included two large plasmids belonging to two *Klebsiella* *pneumoniae* strains (CAV1741 and PMK1). These plasmids were fully assembled and did not share any similarity within the bacterial genome. In contrast, 15 plasmids consistently had a recall value less than 0.9 in all predictions. Four of these fifteen plasmids were not fully covered by SPAdes contigs, precluding complete prediction of the plasmids. The definition of recall per plasmid operated here does not take into account whether plasmids were accurately predicted in unique and independent bins. Programs with a high mean recall (PlasmidSPAdes and cBar, 0.87 and 0.86, respectively) did not predict, or often incorrectly predicted, plasmid binning. cBar performs a binary classification predicting contigs as either ‘plasmid’ or ‘chromosome’, but did not sort the sequences into different plasmids from the same bacterial isolate. PlasmidSPAdes correctly predicted 120 reference plasmids (recall >0.9) present in genomes with more than one reference plasmid (*n*=35). From these 120 correctly predicted plasmids, 19 plasmids were accurately predicted in a single unique bin and 101 plasmids were merged in a bin with other predicted plasmids from the same genome (Supplementary Results 2). Therefore, plasmid binning was not correctly predicted in 84 % of the cases and plasmid structural information was not readily retrievable.

### Prediction per genome

**Fig. 3.**
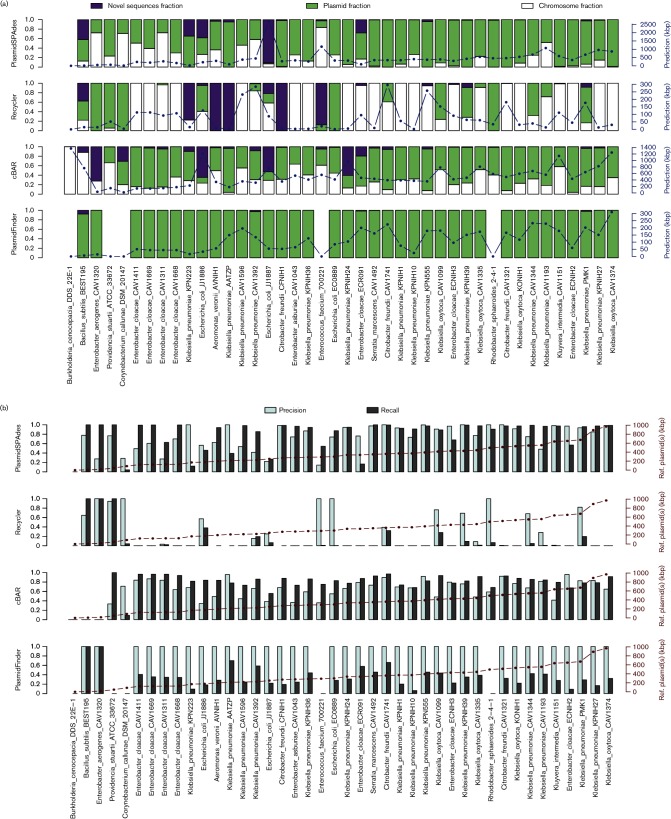
Performance of the programs on a genome level. (a) The prediction of each program was mapped against the reference genomes of each bacterial isolate. Contigs mapping to the reference plasmids were depicted as plasmid fraction (green bars), to the reference chromosome as chromosome fraction (white bars) or to neither as novel sequences fraction (purple bars). On the right-hand-side *y*-axis, the total length (in kbp) of plasmid prediction is indicated. cBar was the only program predicting contigs as plasmids in the genome that was used as a negative control (*Burkholderia* *cenocepacia* DDS 22E-1). (b) Precision and recall values are represented with white and grey bars, respectively. A precision of 1 indicates the absence of contigs mapping to the reference chromosome in the prediction. Recall of 1 indicates the full sequences of all the reference plasmids were present in the prediction. On the right-hand-side *y*-axis, the total plasmid length (in kbp) of a particular bacterial genome is indicated.

Next, performance was evaluated on the genome level; thus, comparing the entirety of all predicted plasmid sequences of each genome against all plasmids of each genome. PlasmidSPAdes analysis resulted in a mean plasmid fraction of 0.72 and a mean chromosome fraction of 0.22 (Fig. 3a). Furthermore, an overall precision of 0.75 and an overall recall of 0.82 were reported. The completeness of the prediction was high even in the bacterial isolates with a high number of reference plasmids. However, PlasmidSPAdes merged plasmid contigs into a single bin if they shared repeated sequences as shown in Fig. S4.

Surprisingly, a mean fraction of 0.06 corresponding to contigs absent from the reference genomes was detected (Fig. 3a and Table S3). Most of the contigs present in the fraction of novel sequences were detected as isolated components by PlasmidSPAdes, with the exception of novel sequences in *Escherichia coli* strains JJ1886 and JJ1887. Predicted plasmid contigs that were absent in the reference genomes of *E. coli* JJ1886 and JJ1887 had high similarity with *Staphylococcus aureus* chromosome and plasmids. This potential contamination was not filtered by PlasmidSPAdes, because its coverage differed from the host chromosome. Further discussion on the identification of potential novel small cryptic plasmids is available in Supplementary Results 3 and 4.

Recycler analysis resulted in a mean plasmid fraction of 0.24, a mean chromosome fraction of 0.62 and a mean fraction of novel sequences of 0.14 (Fig. 3a). We reported an overall precision of 0.30, indicating that a high number of sequences predicted as plasmid originated from the chromosome. Recycler obtained a low overall recall of 0.12 (Fig. 3b). This low value can partly be explained by the fact that the algorithm only reports unique circular sequences. The recall value obtained by Recycler was 1.0 in samples with small or medium size plasmids (e.g. *Bacillus* *subtilis* BEST195 or *Enterobacter* *aerogenes* CAV1320). Furthermore, large plasmids not sharing any repeated sequence with other replicons were also correctly predicted by Recycler (Fig. 3b and Table S4). The Recycler chromosome fraction was further analysed to observe whether non-plasmid mobile genetic elements were predicted. A total of 14 % of the contigs considered as false-positive results and mapping to their respective chromosomes were identified as prophage sequences (Supplementary Results 3, Fig. S5). Recycler more robustly detected plasmid sequences in contaminated samples than PlasmidSPAdes. This feature is reflected in *E. coli* JJ1886 and JJ1887, where the fraction of novel sequences was not higher compared to the rest of the genomes (Fig. 3a). Most of the novel contigs predicted by Recycler were also predicted by PlasmidSPAdes (Table S3). Common features of these novel contigs are a length less than 10 kbp and an intermediate copy number (Supplementary Results 4).

cBar predicted every contig as either plasmid derived or chromosome derived. cBar analysis resulted in a mean plasmid fraction of 0.58, a mean chromosome fraction of 0.33 and a mean fraction of novel sequences of 0.09. We reported an overall precision and recall of 0.62 and 0.76, respectively. However, the precision varied largely across genomes, as reflected in *Providencia* *stuartii* ATCC 33762 (recall=1.0, precision=0.34). This strain has a single plasmid of 48.87 kbp (Fig. S1), which was correctly detected by cBar, but also 19 other contigs (>500 bp) mapping to the chromosome that were wrongly predicted as plasmids (Fig. 3). In *B.* *subtilis* subsp. natto BEST195 and *E.* *aerogenes* CAV1320, which carry single plasmids, precision and recall value were 0 (Fig. 3b). Notably, in the negative control *B.* *cenocepacia* DDS 22E-1, which does not have any plasmids, cBar predicted a total size of 1369 kbp wrongly as plasmid-derived contigs (Fig. 3a). All previously unidentified putative plasmids (Table S3) were also classified as plasmids by cBar, with the exception of two fragments in *Aeromonas* *veronii* AVNIH1 and *Klebsiella* *oxytoca* KONIH1.

PlasmidFinder analysis resulted in a mean plasmid fraction of 0.99 and a mean fraction of novel sequences of 0.01. PlasmidFinder was able to detect at least one plasmid replicon sequence in 37 bacterial strains, but failed to detect any replicon sequence in *Rhodobacter* *sphaeroides* 2-4-1 and in the Gram-positive bacteria *Corynebacterium* *callunae* DSM 20147, *Enterococcus* *faecium* ATCC 700221 and *P. stuartii* ATCC 33672. Surprisingly, in *B.* *subtilis* BEST195, one of the four Gram-positive strains, a recall of 1.0 was obtained. This single plasmid of *B.* *subtilis* BEST195 had an identity of 88 % and covered 82 % of a replicon sequence (NC_015392) from *Salmonella enterica* indexed in the PlasmidFinder database. The database of PlasmidFinder was designed to detect replicon sequences of plasmids from the Enterobacteriaceae. Therefore, we excluded all Gram-positive genomes to calculate the overall precision and recall of PlasmidFinder. This resulted in an overall precision of 1.0, indicating that no false-positive sequences were predicted as plasmids. However, the low overall recall of 0.36 was due to the low connectivity of the assemblies that were generated using only short-read sequencing data (Fig. 3b).

### Conclusion

The large majority of plasmids (89.9 %) could be correctly predicted by one of the tested programs. However, in many cases, the predictions were fragmented (all programs), contaminated by chromosome sequences (cBar, Recycler, PlasmidSPAdes), the binning of the plasmids were unclear (cBar, PlasmidSPAdes) and the plasmids were incomplete (all programs). In absence of reference plasmid sequences, disentangling or binning the sequences into separate plasmids is a challenging step.

PlasmidSPAdes fully or partially predicted most plasmids (recall=0.82). The major drawback of PlasmidSPAdes was the merging of predicted plasmids into a single bin. This limitation can partially be overcome by a similar methodology as previously applied in PLACNET [6]. By visualizing the plasmid graph and connecting contigs with a similar coverage and scaffolding linkage, plasmid sub-graphs can be separated manually, but only if the different plasmids sufficiently differ in their copy number [10] (Fig. S3). Repeat sequences, such as transposases, that merge different components in the assembly graph, can be spotted by their high number of scaffolding links and coverage. However, this process is highly dependent on the expertise of the individual analysing the data, may be difficult to reproduce independently, and can only be performed if coverage of plasmids differs. Consequently, this approach limits the high-throughput analysis of short-read WGS data to correctly predict plasmid sequences.

Recycler applies an innovative and general approach to plasmid prediction, and successfully extracted complete plasmid sequences if they had circular features present in the assembly graph. Most large plasmids, however, tend to be assembled into several contigs due to the presence of repeated sequences with high coverage. Recycler failed to extract these types of plasmids and in many cases only extracted non-plasmid mobile elements. cBar was originally designed to categorize chromosome and plasmids in metagenomic sequences. Its accuracy is known to be lower for long plasmids because the nucleotide composition of these plasmids is similar to the host chromosome [14]. However, the overall recall of cBar is high (0.78) and it might be well-suited to confirm if a sequence was predicted to be plasmid-derived by another method.

The results of PlasmidFinder indicated a high reliability of the prediction. If applied to PlasmidSPAdes predictions, the detection of different incompatibility groups by PlasmidFinder could either indicate the presence of two or more plasmids merged together into a single component or the presence of a multireplicon plasmid.

To obtain the full sequences of plasmids, long-read sequencing data can be a solution [15]. However, to our surprise, PlasmidSPAdes and Recycler predicted a substantial number of contigs (fraction of novel sequences: 0.06 and 0.14, respectively) that were not present in the complete reference genomes sequenced with long reads. These sequences could originate from sequences filtered as contaminants, but could also represent small replicons (Supplementary Results 2 and 4). As described elsewhere, the hierarchical genome assembly process (HGAP) can lead to missing small plasmids in the main assembly when using a seed read length cut-off greater than actual plasmid size [16, 17]. Library preparation withDNA size selection prior to PacBio sequencing can also obviate small plasmids when the cut-off selected is higher than actual replicon size [2].

We showed that it is possible to automatically predict the sequences of small and circular plasmids. Nonetheless, the correct prediction of large plasmids (>50 kbp) containing repeated sequences remains challenging using short-read sequencing data only.

## Data bibliography

1. Arredondo-Alonso S, Willems, RJ, van Schaik, W. Schürch AC. GitLab repository, https://gitlab.com/sirarredondo/Plasmid_Assembly.git (2016).

## Supplementary Data

426 Supplementary File 1

## Supplementary Data

35 Supplementary File 2

## Supplementary Data

28 Supplementary File 3
