# Supplementary material for: On the (im)possibility of reconstructing plasmids from whole-genome short-read sequencing data
Source: Microb Genom. 2017 Aug 18;3(10):e000128. doi: 10.1099/mgen.0.000128 (PMC5695206; doi:10.1099/mgen.0.000128)
Supplement: Supplementary File 1 [file mgen-3-128-s001.pdf]

Supplementary Data

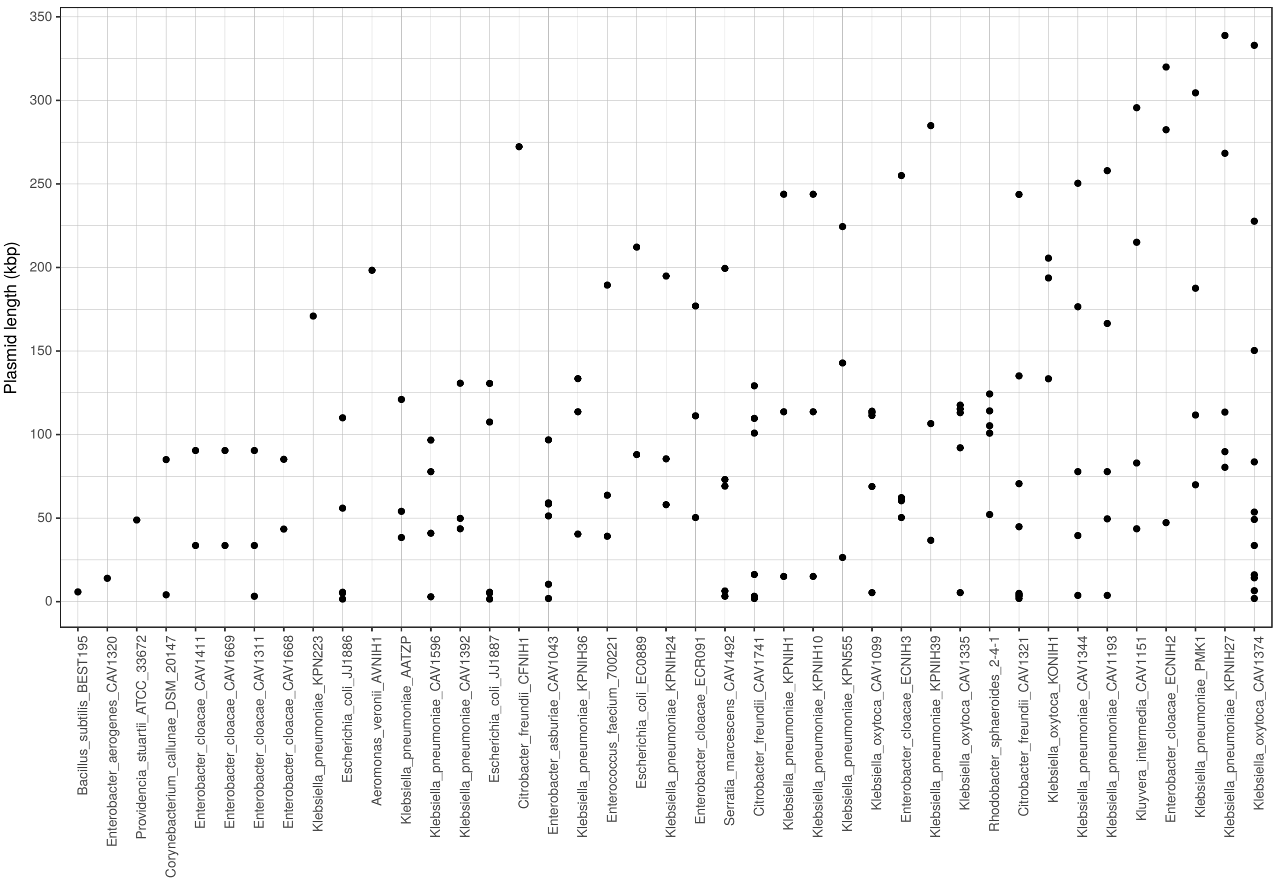

Figure S1: **Overview of reference plasmids.** The size of the reference plasmids is shown for each bacterial isolate. Strains were sorted based on their total plasmid length. *K. oxytoca* strain CAV1374 was the most complex isolate with eleven plasmids ranging from 1.9 to 332.9 kbp.

Table S1: Bacterial genomes included in this study.

.

**Genome**	**SRA**	**Genome accession**	**Number of plasmids**	**Range size**	**Total length**
Burkholderia cenocepacia strain DDS 22E-1	SRR1618480	GCA_000755725.1	0	-	0
Bacillus subtilis subsp. natto BEST195	DRR016448	GCA_000209795.2	1	5.8	5.8
Enterobacter aerogenes strain CAV1320	SRR2965748	GCA_001021995.1	1	13.9	13.9
Providencia stuartii strain ATCC 33672	SRR1558174	GCA_000754345.1	1	48.8	48.8
Corynebacterium callunae DSM 20147	SRR892039	GCA_000420585.1	2	4.1-85.0	89.132
Enterobacter cloacae strain CAV1411	SRR2965820	GCA_001022075.1	2	33.6-90.4	124.0
Enterobacter cloacae strain CAV1669	SRR2965616	GCA_001022255.1	2	33.6-90.4	124.0
Enterobacter cloacae strain CAV1311	SRR2965815	GCA_001022015.1	3	3.2-90.4	127.2
Enterobacter cloacae strain CAV1668	SRR2965612	GCA_001022055.1	2	43.4-85.1	128.6
Klebsiella pneumoniae strain Kpn223	SRR3465557	GCA_001663435.1	1	170.9	170.9
Escherichia coli JJ1886	SRR933487	GCA_000493755.1	5	1.5-110.0	178.3
Aeromonas veronii strain AVNIH1	SRR3465535	GCA_001634325.1	1	198.3	198.3
Klebsiella pneumoniae strain AATZP	SRR3228444	GCA_001648215.1	3	38.3-121.0	213.4
Klebsiella pneumoniae strain CAV1596	SRR1582868	GCA_001022235.1	4	2.9-96.7	218.3
Klebsiella pneumoniae strain CAV1392	SRR1582895	GCA_001022035.1	3	43.6-130.7	224.1
Escherichia coli JJ1887	SRR933489	GCA_001593565.1	5	1.5-130.6	250.4
Citrobacter freundii CFNIH1	SRR1284629	GCA_000648515.1	1	272.2	272.2
Enterobacter asburiae strain CAV1043	SRR2965752	GCA_001022095.1	6	1.9-96.8	278.0
Klebsiella pneumoniae strain KPNIH36	SRR3222156	GCA_001675125.1	3	40.44-133.4	287.5
Enterococcus faecium strain ATCC 700221	SRR3176159	GCA_001594345.1	3	39.1-189.4	292.2
Escherichia coli strain ECO889	SRR3465539	GCA_001663475.1	2	88.0-212.1	300.2
Klebsiella pneumoniae subsp. pneumoniae KPNIH24	SRR1501128	GCA_000714675.1	3	58.0-194.8	338.4
Enterobacter cloacae ECR091	SRR1576808	GCA_000750275.1	3	50.3-176.9	338.5
Serratia marcescens strain CAV1492	SRR2965730	GCA_001022215.1	5	3.2-199.4	351.3
Citrobacter freundii strain CAV1741	SRR2965739	GCA_001022275.1	6	1.9-129.1	361.1
Klebsiella pneumoniae subsp. pneumoniae KPNIH1	SRR1505904	GCA_000281535.2	3	15.0-243.8	372.5
Klebsiella pneumoniae subsp. pneumoniae KPNIH10	SRR1427234	GCA_000281435.2	3	15.0-243.8	372.5
Klebsiella pneumoniae strain Kpn555	SRR3465562	GCA_001663455.1	3	26.4-224.4	393.7
Klebsiella oxytoca strain CAV1099	SRR2965639	GCA_001022295.1	5	5.4-113.9	412.8
Enterobacter cloacae ECNIH3	SRR1576778	GCA_000750225.1	4	50.3-255.0	427.9
Klebsiella pneumoniae strain KPNIH39	SRR3217430	GCA_001663295.1	3	36.7-284.8	428.1
Klebsiella oxytoca strain CAV1335	SRR2965660	GCA_001022115.1	5	5.4-117.6	443.5
Rhodobacter sphaeroides 2.4.1	SRR522246	GCA_000273405.1	5	52.1-124.3	496.7
Citrobacter freundii strain CAV1321	SRR2965690	GCA_001022155.1	9	1.9-234.7	512.4
Klebsiella oxytoca KONIH1	SRR1501122	GCA_000714655.1	3	133.3-205.5	532.7
Klebsiella pneumoniae strain CAV1344	SRR1582875	GCA_001022175.1	5	3.7-250.3	547.9
Klebsiella pneumoniae strain CAV1193	SRR2965672	GCA_001456135.1	5	3.7-257.9	555.5
Kluyvera intermedia strain CAV1151	SRR2965721	GCA_001022135.1	4	43.6-295.6	637.7
Enterobacter cloacae ECNIH2	SRR1515967	GCA_000724505.1	3	47.2-319.9	649.7
Klebsiella pneumoniae strain PMK1	SRR1508819	GCA_000764615.1	4	69.9-304.5	673.7
Klebsiella pneumoniae subsp. pneumoniae KPNIH27	SRR1427243	GCA_000695935.1	5	80.4-338.8	890.8
Klebsiella oxytoca strain CAV1374	SRR2965655	GCA_001022195.1	11	1.9-332.9	969.8

Supplementary Methods: Evaluation metrics

We predicted plasmids from short reads with four different programs: PlasmidFinder, cBar, Recycler and PlasmidSPAdes. Reads were downloaded from the SRA database using the sra-toolkit and subsequently trimmed using seqtk with the command 'trimfq'. This trimmed low-quality bases from both ends using the Phred algorithm, which uses base error probabilities calculated from the phred quality values. We selected an error probability cutoff value of 0.05. *De novo* assembly was performed using SPAdes 3.8.2 on a high performance computer running CentOS7. For each sample, the assembly graph and resulting contigs corresponding to the maximum *k-mer* used by SPAdes 3.8.2 were selected [1]. Contigs with a size less than 500 bp were filtered out.

- *PlasmidFinder.* To replicate results that would be obtained through the use of the PlasmidFinder web interface, we downloaded the PlasmidFinder database containing 121 replicon sequences (updated on 16 March 2016) from the Center for Genomic Epidemiology (https//cge.cbs.dtu.dk//services/data.php). We then performed nucleotide BLAST (NCBI-BLAST version 2.2.28+) searches against this database [2]. Contigs were identified as plasmids if they had a minimum identity of 80% and covered at least 60% of the replicon sequence, consistent with the parameters used to identify plasmids in bacterial whole-genome data by the authors of PlasmidFinder [3]. Contigs in which a replicon sequence was identified were considered as PlasmidFinder prediction.

- *cBar.* We downloaded cBar version 1.2 from http://csbl.bmb.uga.edu/ ffzhou/cBar/cBar.1.2.tar.gz and used it to categorize contigs derived by SPAdes 3.8.2. Contigs categorized as plasmid-derived were considered as cBar prediction.

- *Recycler.* We downloaded Recycler (single version, date: 07-03-2016) from https://github.com/Shamir-Lab/Recycler. The BAM file required as input by Recycler was created by alignment of the trimmed reads against the resulting contigs using Bwa 0.7.12 [4] and samtools 1.3.1 [5]. Cycles reported in the assembly graph were considered as Recycler prediction.

- *PlasmidSPAdes.* We run PlasmidSPAdes (packaged in SPAdes 3.8.2) with standard parameters. The components reported in contigs.fasta were considered as PlasmidSPAdes prediction.

Measures for the evaluation

We evaluated the performance of each program regarding accuracy and completeness. Quast (version 4.1) [6] was used to assign each of the predicted contigs to one of the following three categories: *Plasmid, chromosome* or *novel sequences fraction.* Quast was run with the following command to map the prediction against the reference genome (chromosome and plasmid(s)) for each genome project:

python quast.py contigs.fasta -R all_references.fasta -o quast_analysis_genome –min-alignment 500 –ambiguity-usage all

Quast takes as default a minimum identity alignment (IDY%) of 95%, all the alignments with less than this threshold are thus discarded. A minimum alignment of 500 bp was considered to assign a contig to a specific genome. We defined the argument "all" in the flag –ambiguity-usage to report all equally good alignments of a contig (e.g. a transposase present in the chromosome and in a reference plasmid). Equally good alignments are defined in Quast using the following definition:

'all alignments are sorted by decreasing LENxIDY% value. All alignments with LENxIDY% less than Sxbest(LENxIDY%) are discarded. S should be between 0.8 and 1.0. The default value is 0.99.'

Accordingly, we encountered different scenarios:

- **Contigs with a single alignment to a reference sequence** (most frequent). These contigs could be directly assigned to: *Plasmid fraction* (true positive result) or *Chromosome fraction* (false positive result).

- **Contigs without any significant alignment against the reference genome.** These contigs were assigned to the *fraction of novel sequences.* These contigs can be the result of contamination during a sequencing project as it is apparent in *Escherichia coli* JJ1886 and JJ1887. But, some contigs corresponded to small plasmids not present in the PacBio assemblies as further elaborated in Supplementary Results and Table S3.

- **Contigs mapping to the chromosome and to a reference plasmid(s). This scenario could be further divided in two cases:**

Contigs with repeated sequences e.g. transposases which are present in the chromosome but also in a reference plasmid. In the Quast output, we observed two alignments (same length and score mapping) to two different sequences. In these cases, we assigned these contigs only to the *Plasmid fraction*. We considered these contigs only as true positive results because all the base pairs from the contig were present in a reference plasmid.

Contigs containing missamblies. A missassembly corresponds to cases where different regions of the same contig are mapping to different locations of a reference genome(s). Quast defines different kind of missamblies: local missassemblies, relocations and translocations. Local missassemblies and relocations correspond to contigs where the left and right flank are mapping to different parts of the same reference sequence. Therefore, contigs can be assigned either to *Plasmid fraction* or *Chromosome fraction* because the missassembly occurs in the same reference sequence. For precision calculation purposes, we only decided to filter out contigs with a translocation event between the chromosome and a reference plasmid. This means, a single contig had two parts (left and rigth flanks; with a minimum alignment of 500 bp and passing Quast score) mapping e.g. left flank to the chromosome and right flank to a reference plasmid. In these cases, we could not assigne the whole contig to either the *Plasmid fraction* or the *Chromosome fraction* and we removed these contigs to calculate precision.

Icarus (packaged in Quast 4.1) [7] was used to visualize the alignments between the reference genomes and the predicted sequences.
We defined the previously introduced terms as:

- **Plasmid fraction**. Fraction of the prediction that matched the reference plasmids (true positive prediction).
- **Chromosome fraction**. Fraction of the prediction that matched the reference chromosome (false positive prediction). This fraction can include non-plasmid mobile genetic elements from the chromosome such as phages or transposable elements.
- **Fraction of novel sequences**. Fraction of the prediction not mapping to either the reference plasmid or the chromosome, thus corresponding to contigs absent from the reference assembly.

The programs were further evaluated using the following metrics.

- **Recall** was defined as the fraction of the reference plasmid(s) covered by the prediction. On the individual plasmid level, a recall of 1 indicates that the full sequence of the reference plasmid was present among the predicted plasmids. On the whole genome level, a recall of 1 indicates all reference plasmids were fully present among the predicted plasmids. However, recall does not consider whether predicted plasmid contigs were correctly binned.

Recall value was estimated using the genome fraction reported in Quast.

- **Precision**. We defined precision as:

$$\frac{Plasmid\ fraction}{Plasmid\ fraction + Chromosome\ fraction} \tag{1}$$

The *fraction of novel sequences* was ignored when calculating precision. Total contig length was considered to estimate recall and precision.
For each genome project (n=42) we calculated precision and recall values. To calculate the overall precision and recall of PlasmidSPAdes, Recycler and PlasmidFinder we excluded the negative control *B. cenocepacia* strain 22E-1. The overall precision and recall of cBar was calculated considering *B. cenocepacia* strain 22E-1 as the program detected a high number of contigs corresponding to false positive results (Supplementary Text 1). Finally, the overall precision and recall of PlasmidFinder was calculated filtering out all the genome projects from gram-positive bacteria.
Scaffold linkage of specific contigs in the PlasmidSPAdes assembly graph of a selection of genomes was visualized with Bandage (version 0.7.1) [8].

The workflow (Figure S2) was written in bash and python (version 2.7) and subsequent analysis done in R (version 0.99.982).

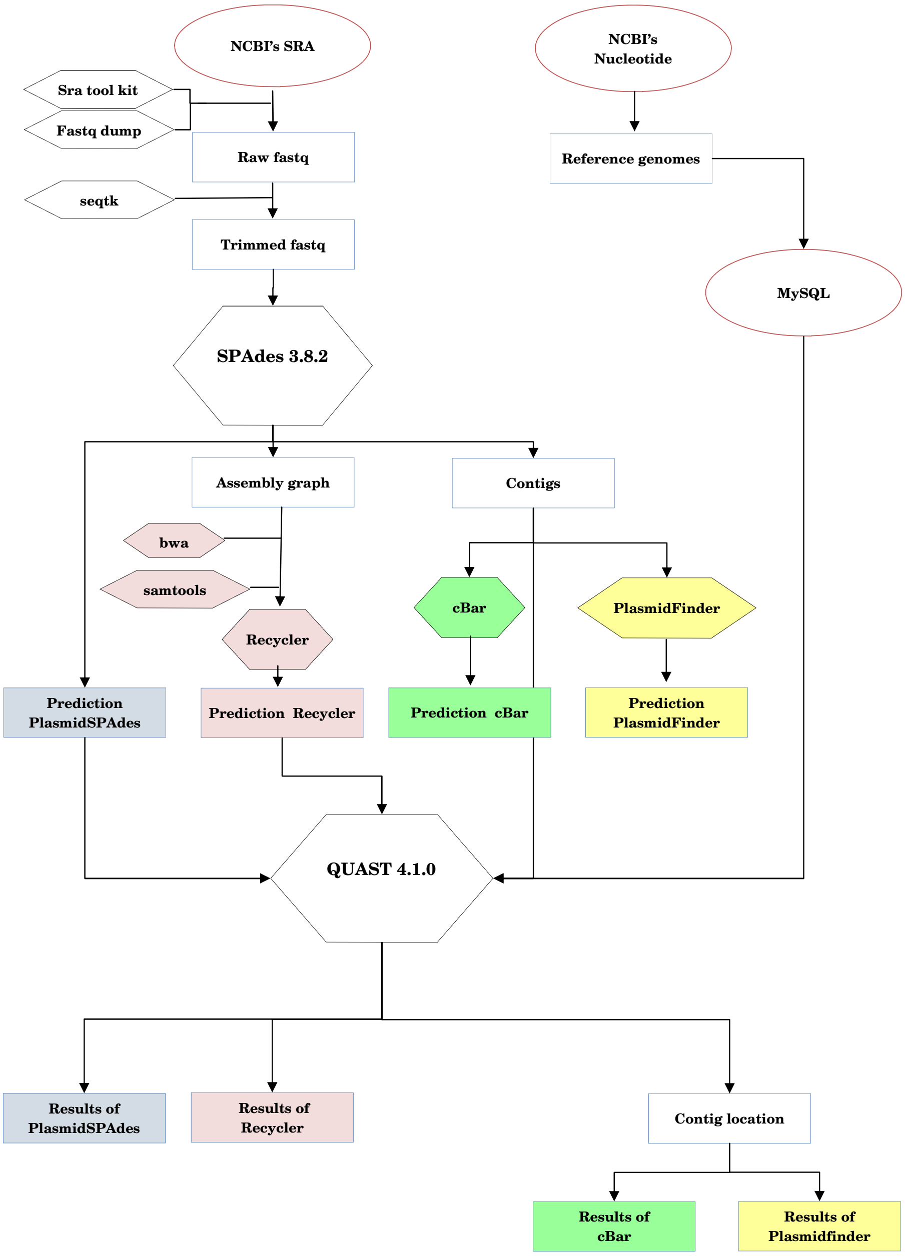

Figure S2: Analysis Workflow. Diagram representing the analysis reported in the manuscript.

Supplementary Results 1 Detailed description of genomes considered as positive controls

The following genomes were previously analyzed by the authors of PlasmidSPAdes and Recycler to validate their algorithms [9, 10].

Escherichia coli JJ1886

Recycler predicted the sequence of seven possible plasmids from *E. coli* JJ1886. Four of them corresponded to the reference plasmids whereas three sequences did not map either the plasmid references nor the chromosome of *E. coli* JJ1886. These three sequences were confirmed as plasmids by nucleotide BLAST although no other evidence of plasmid-related genes were found in the annotation by Prokka. In addition, we found two sequences of 42.6 kbp and 8.2 kbp corresponding to the chromosome of *E. coli* JJ1886. A best blast hit of the sequence with 42.6 kbp mapping to the chromosome suggested a phage origin.

PlasmidSPAdes was able to recover a fraction of the plasmid pJJ1886_5, but plasmids pJJ1886_1 and pJJ1886_3 were not detected. Five components did not map to either the chromosome or the reference plasmids which suggested that they were putative unidentified plasmids. The components corresponding to *S. aureus* plasmids were present in a copy number of less than 1. Frequently, short-read length plasmids are in high copy number to ensure their prevalence in the next generations [11]. These findings suggested these novel sequences identified in *E. coli* JJ1886 could constitute contamination during the library preparation. PlasmidSPAdes did not remove some parts of the chromosome from *S. aureus* because its coverage differed from *E. coli* JJ1886.

cBar identified several plasmid sequences as chromosomal resulting in a low precision (Table S2). However, it was the program with the best recall value (0. 83) because it recovered 12 contigs (>500 bp) belonging to pJJ1886_5. PlasmidFinder detected the presence of two plasmid replicon initiator sequences corresponding to the incompatibility group IncF. Both replicons are located in the plasmid pJJ1886_5 of the contigs with a size of 8.0 kbp and 12.9 kbp.

Citrobacter freundii CFNIH1

PlasmidSPAdes detected a component with a length of 275.6 kbp, composed by 19 contigs (>1 kbp) that matched the reference plasmid pKEC-a3c. In addition, a second component composed by a single contig of 5.4 kbp and an inferred copy number of 14.1 was identified. Recycler was not able to recover the plasmid pKEC-a3c (Table S2). However, it also extracted the same novel component of 5.4 kbp with a coverage ratio of 14.1. We performed a dot-plot of the sequence against itself to observe the presence of circularization signatures at the ends. The sequence had a best blast hit corresponding to "*Klebsiella oxytoca* strain CAV1335 plasmid pCAV-1335-5410, complete sequence" with a length of 5.4 kbp. Annotation made by Prokka identified the presence of mobilization protein MbeC and relaxase MbeA.

The sequence of 5.4 kbp predicted by PlasmidSPAdes and Recycler is the same with a slight difference. Recycler extracted one of the repeat sequences present at the end of the contigs obtaining a final plasmid sequence of 5410 bp. However, PlasmidSPAdes extracted the plasmid sequences without removing one of the repeats. The previous findings suggested the presence of a complete plasmid sequence of 5.4 kbp which was not previously reported in *C.freundii* CFNIH1.

PlasmidFinder detected the presence of two replication initiator proteins present in pKEC-a3c. The replicon sequences corresponded to the incompatibility groups IncN and IncA.

Corynebacterium callunae DSM 20147

PlasmidSPAdes detected two components of 10.4 kbp and 4.2 kbp. A low precision value was obtained because the component of 10.3 kbp was composed by a single contig mapping to the chromosome (Table S2). The component of 4.2 kbp corresponded to the reference plasmid pCC1. Recycler detected exclusively the reference plasmid pCC1 whereas no false positive results were obtained.

cBar obtained a low recall value because only one contig corresponding to pCC2 was correctly identified as plasmid (Table S2). PlasmidFinder was not able to locate any replication initiator sequence in the two reference plasmids present in *C. callunae* DSM 20147. The database of PlasmidFinder was constructed using replicon sequences from the family *Enterobacteriaceae*. Replicon sequences from Gram positive bacteria may differ and may explain the lack of true positive results for this genome.

Rhodobacter sphaeroides 2.4.1

PlasmidSPAdes was able to detect a large component of 458 kbp including the five reference plasmids. However, the program was not able to separate the plasmids in different components. PlasmidSPAdes merged them in a

single component due to the presence of repeated sequences frustrating the detection of each plasmid as different sub graphs. Visualization of the plasmid graph using Bandage spotted one contig containing a transposase shared in the different physical DNA units (Figure S3). Recycler was only able to detect small fractions from plasmid Ax and plasmid D whereas lack of false positive results were reported (Table S2). PlasmidFinder did not detect any plasmid replicon sequences.

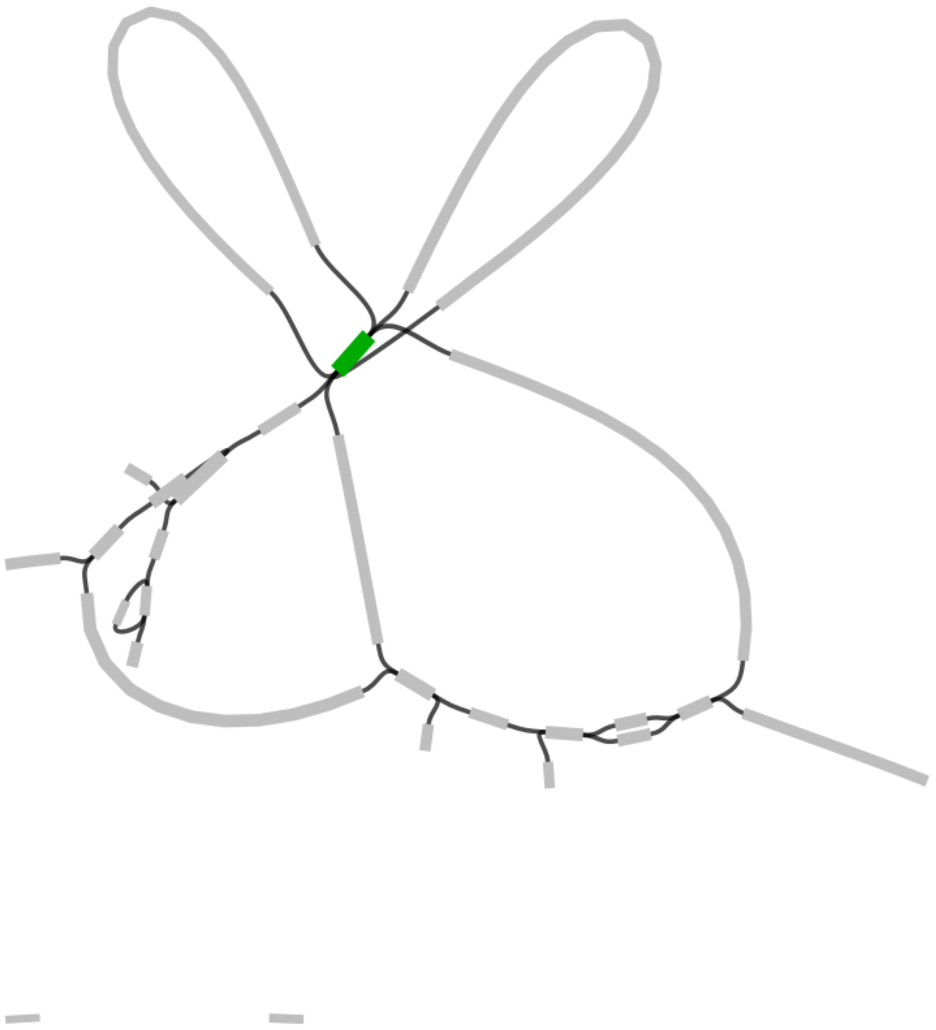

Figure S3: **Bandage representation of the assembly graph generated by PlasmidSPAdes in R. sphaeroides 2.4.1.** Green contig with a length of 1.6 kbp and coverage of 536 was identified as a transposase by BLASTx.

Burkholderia cenocepacia DDS 22E-1

This genome does not contain any reference plasmid but it is composed by three chromosomes with a size of 1.16 Mbp, 3.20 Mbp and 3.66 Mbp. PlasmidSPAdes and Recycler did not detect any plasmid sequence thus the outcomes of both programs corresponded to empty files. Additionally, PlasmidFinder did not find any replicon sequence within the chromosomes of *B.cenocepacia* DDS 22E-1. cBar predicted 1481 contigs (>500 bp) wrongly as plasmid-derived sequences.

Table S2: Precision and recall of each program in the genome projects considered as positive controls

**Strain**	**Program**	**Precision (%)**	**Recall (%)**
E. coli JJ1886	pSPAdes	0.56	0.46
	Recycler	0.57	0.38
	PlasmidFinder	1.00	0.18
	cBar	0.34	0.84
C. freundii CFNIH1	pSPAdes	0.99	0.99
	Recycler	0.00	0.00
	PlasmidFinder	1.00	0.19
	cBar	0.68	0.88
C. callunae DSM 20147	pSPAdes	0.28	0.04
	Recycler	1.00	0.04
	PlasmidFinder	0.00	0.00
	cBar	0.71	0.09
R. sphaeroides 2-4-1	pSPAdes	1.00	0.91
	Recycler	1.00	0.07
	PlasmidFinder	0.00	0.00
	cBar	0.59	0.69

Supplementary Results 2 PlasmidSPAdes structural report

The major pitfall of PlasmidSPAdes was the erroneous assignment of predicted plasmid contigs belonging to different reference plasmids into the same bin. To visualize and explain this issue we selected only genome projects with more than one reference plasmid (n=35). From these genomes, only plasmids correctly predicted by PlasmidSPAdes (n=120, recall >0.9), have been considered. For each reference plasmid we observed whether it was merged with another predicted plasmid from the same genomes. This resulted in 19 plasmids predicted in a single unique bin and 101 plasmids merged together with other predicted plasmids. Therefore, 84.2% of the well predicted plasmids were merged together with other plasmids from the same genome project.

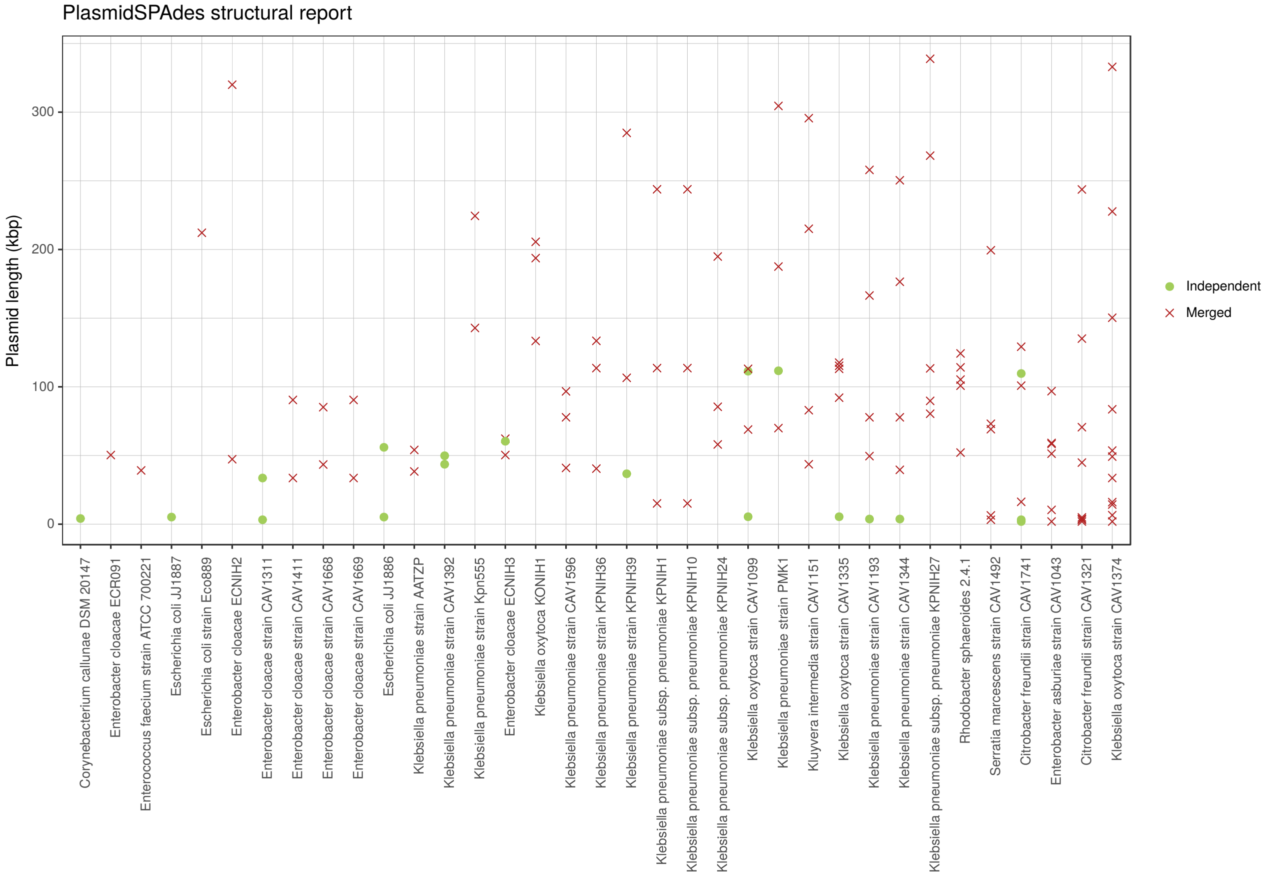

Figure S4: **PlasmidSPAdes structural report.** Plasmids correctly predicted by PlasmidSPAdes (recall >0.9) were further evaluated to assess if they were merged in a single unique bin with other reference plasmids. Only genome projects with more than one reference plasmid (n=35) have been considered.

Supplementary Results 3 Recycler chromosome fraction analysis

Recycler was designed to extract circular sequences from the assembly graph. Therefore, Recycler predictions also contained non-plasmid mobile genetic elements with a potential circularization signature. We further elaborated on this extracting all the contigs (n=94) which were part of the *chromosome fraction* and annotated them using Prokka. First we checked the presence of genes annotated with the following keywords to assess if there were contigs with potential phage-related genes.

*grep -i -E ”—capsid—head—integrase—plate—tail—fiber—coat—phage—transposase—portal—terminase—protease—lysin” *.tbl*

This allowed us to identify 22 contigs with potential phage-related genes. Furthermore, we used Phaster (PHAge Search Tool Enhanced Release), a program specially focused on the identification and annotation of (pro)phage sequences [12]. From 22 contigs with potential-phage related genes, only 13 were identified as prophages. Therefore, 14% (13/94) of the contigs classified as false positives and assigned to the *chromosme fraction* were identified as prophages sequences.
Furthermore, we observed the same phage sequence was extracted in genomes belonging to the same *species*. For example, we highlight the phage sequence of 41.9 kbp predicted by Recycler in *E. cloacae* strain CAV1311, *E.cloacae* strain CAV1411, *E.cloacae* strain CAV1668 and *E.cloacae* strain CAV1669 (Figure S5).

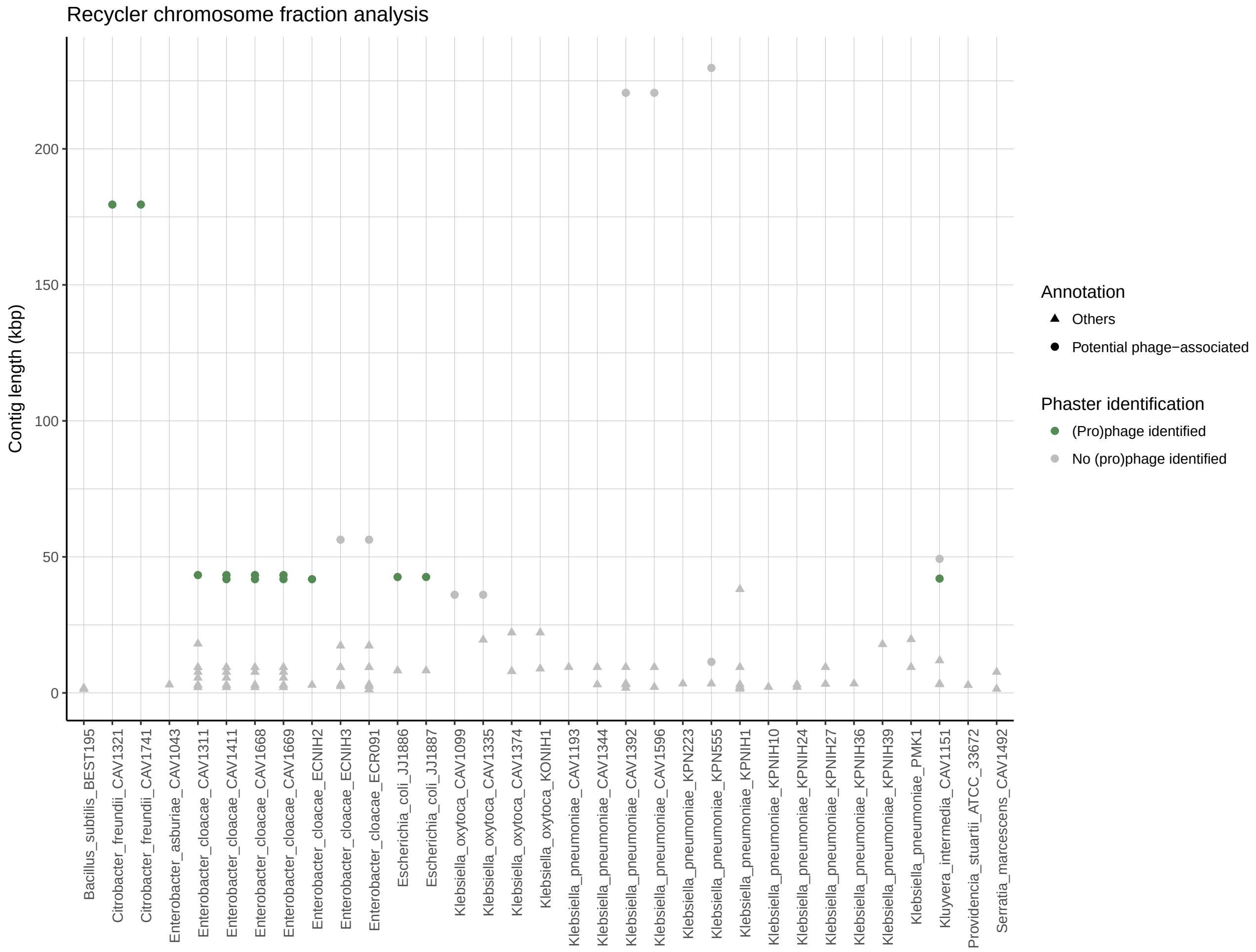

Figure S5: **Recycler chromosome fraction analysis.** Contigs assigned to the chromosome fraction were annotated by Prokka and queried against Phaster to observe phage sequences. Strains were sorted alphabetically (x-axis) to group genome projects belonging to the same *species*.

Supplementary Results 4 Components not mapping to the reference genomes

In this section we describe potential novel plasmids detected by PlasmidSPAdes and Recycler as plasmid components in the graph. Only components with a single contig and exceeding a minimum length of 1000 bp were analyzed.
Novel contigs were further analyzed and annotated using Prokka (version 1.12-beta)[13]. To identify potential novel plasmids we compared these sequences to the non-redundant nucleotide database of the NCBI using BLAST. The best blast hit was extracted selecting minimum e-value and highest bit-score as previously described [10]. The completeness of the potential novel mobile genetic elements was corroborated by generating a dot-plot aligning the sequence to itself [14]. The presence of the same repeated sequence at the ends of the contig suggested a potential circularization signature. We also considered the k-mer coverage ratio as a feature to identify potential small plasmids. Each contig reported in SPAdes 3.8.2 has an associated k-mer coverage, defined as $c\kappa = c * (l - k + 1)/l$, where $c\kappa$ = k-mer coverage, c = nucleotide coverage, l = read length and k = k-mer length. We considered the median coverage reported by PlasmidSPAdes as an estimation of the chromosome coverage. Finally, the reported k-mer coverage ratio corresponds to the k-mer coverage of a novel contig divided by its respective median coverage. This analysis is summarized in Table S3.

Bacillus subtilis subsp. natto BEST195

PlasmidSPAdes and Recycler identified a single component not mapping to the reference assembly with a length of 5386 bp, present in a k-mer coverage ratio of 10 and with circularization signatures.

Klebsiella pneumoniae strain Kpn223

PlasmidSPAdes identified two isolated components formed by a single contig with a length of 4.29 and 4.14 kbp. Recycler identified the same sequences but excluded one of the adjoining regions and detected another component not mapping to the reference of 3478 bp. The sequence of 4167 bp had as best blast hit "*Klebsiella pneumoniae* strain 0773 plasmid pKpn114, complete sequence" with a length of 4.21 kbp. The sequence of 4014 bp did not have any significant blast hit even though Prokka detected the presence of a Mobilization protein A and circularization signatures were present. In addition, the sequence identified by Recycler with a length of 3478 bp had a best blast hit corresponding to "*Enterobacter sp.* FY-07 plasmid pAKI40B, complete sequence". However, the k-mer coverage ratio suggested it is necessary to validate experimentally these novel plasmids to confirm them as stable residents in the host.

Escherichia coli JJ1886

PlasmidSPAdes identified two components composed by a single contig not mapping to the reference assembly. The component with a length of 11.10 kbp had as best blast hit "*Staphylococcus aureus* subsp. aureus strain LA-MRSA ST398 isolate E154, complete genome". As explained in Supplementary Text 1, PlasmidSPAdes recovered part of the chromosome of *S. aureus* because its coverage differs from the host chromosome. The second isolated component identified by PlasmidSPAdes was also present in Recycler prediction with a length of 1.6 kbp. This component had as best blast hit "Staphylococcus aureus strain C2355 plasmid pUR2355, complete sequence" with a length of 7.6 kbp.

Additionally, Recycler predicted two other isolated components with a length of 2361 and 2216 bp. Both components had best blast hits corresponding to "*Staphylococcus aureus* subsp. aureus strain LA-MRSA ST398 plasmid plinE154, complete sequence and "*Staphylococcus sp.* plasmid pST94 qacG and rep94 genes".

The presence of *S. aureus* chromosome in PlasmidSPAdes prediction and a k-mer coverage ratio below 1.0 suggests this sample was contaminated with *S.aureus* DNA [9].

Aeromonas veronii strain AVNIH1

PlasmidSPAdes identified two components formed by a single contig not mapping to the reference assembly of *A. veronii* strain AVNIH1. In addition, Recycler also identified the same components and it extracted one of the repeated sequences present at both ends of the contigs. The largest sequence had as best blast hit "*Aeromonas salmonicida* subsp. salmonicida strain JF2507 plasmid pAsal1D, complete sequence" with a length of 9.1 kbp. The other sequence corresponded to "Uncultured prokaryote from Rat gut metagenome metamobilome, isolate RGRH0694" with a length of 1.8 kbp. The presence of circularization signatures, a similar best blast hit length corresponding to previous report plasmids and the inferred plasmid copy numbers suggested the presence of two small complete plasmids not reported with a length of 7114 bp and 1736 bp.

Klebsiella pneumoniae strain AATZP

PlasmidSPAdes identified an isolated component formed by a single contig of 4.29 kbp. Additionally, Recycler did not identify any reference plasmid present in *K. pneumoniae* strain AATZP but it also detected the same putative unidentified plasmid. Best blast hit corresponded to: "*Klebsiella pneumoniae* subsp. pneumoniae Kp13 plasmid pKP13c, complete sequence" with a length of 5.06 kbp. A similar blast hit length and the presence of circularization signatures at both ends of the sequence suggested the identification of a small cryptic plasmid with a length of 4167 bp not present in the reference assembly of *K. pneumoniae* strain AATZP.

Klebsiella pneumoniae strain CAV1392

PlasmidSPAdes identified an isolated component composed by a single contig of 2.57 kbp with a k-mer coverage ratio of 0.1. Additionally, Recycler identified the same component and reporting the correct size (2495 bp). Best blast hit corresponded to "*Enterobacter sp.* W001 plasmid pR23, complete sequence" with a length of 10.49 kbp. The presence of circularization signatures at the end suggested the completeness of the plasmid.

Citrobacter freundii CFNIH1

PlasmidSPAdes and Recycler identified an isolated component with a length of 5.4 kbp and with a k-mer coverage ratio of 14.1. Dot-plot of the contig to itself confirmed the presence of circularization. Furthermore, best blast hit corresponded to "*Klebsiella oxytoca* strain CAV1335 plasmid pCAV-1335-5410, complete sequence" with a length of 5.4 kbp. Annotation made by Prokka identified the presence of relaxase MbeA. This novel plasmid had already been detected in PlasmidSPAdes original publication [10].

Enterococcus faecium strain ATCC 700221

Both programs identified the presence of two components not mapping to the reference assembly with a length of 12462 bp and 5386 bp. The largest contig had as best blast hit "*Enterococcus faecium* plasmid p200B" with a length of 12.5 kbp. The sequence of 5386 had as best blast hit "Enterobacteria phage phiX174, complete genome" with the same length. Additionally, in both cases there was presence of circularization signatures.

The presence of phage sequences was common in the report given by Recycler and PlasmidSPAdes due to the presence of circularity signatures and differences in the coverage with the host chromosome. The above findings suggest the identification of a novel plasmid with a length of 12462 bp.

Klebsiella pneumoniae strain Kpn555

PlasmidSPAdes and Recycler identified the same components not mapping to the reference assembly. The sequence of 4048 bp had as best blast hit "*Escherichia coli* strain EC19 plasmid pEC19-1 hypothetical proteins, MobA, MobB, and MobC genes, complete cds" with a length of 4.86 kbp. The sequence with a length of 3478 bp had as best blast hit "*Klebsiella pneumoniae* subsp. pneumoniae MGH 78578 plasmid pKPN7, complete sequence" with a length of 3.78 kbp.

Finally, two sequences with a length of 2874 bp and 2798 bp were also reported. In both cases, the best blast hit corresponded to "Uncultured bacterium extrachromosomal DNA RGI00802" with a length of 2.80 kbp.

Sequence annotation and circularization signatures indicated the presence of four small novel plasmids but their k-mer coverage ratio suggests experimental validation is required to discard these components as possible contamination.

Klebsiella pneumoniae strain PMK1

PlasmidSPades and Recycler identified the same three components not present in the reference assembly.

Contig annotation spotted the presence of plasmid-genes related in the sequence of 5640 bp. Best blast hit corresponded to "Uncultured prokaryote from Rat gut metagenome metamobilome, plasmid pRGRH1815" with a length of 7.10 kbp. The presence of circularization signatures and a high k-mer coverage ratio suggested the characterization of a plasmid with a length of 5640 bp.

In addition, the sequence with a length of 3770 bp presented circularization signatures and a best blast hit corresponding to "*Escherichia coli* strain NCTC 9034 plasmid pEC34A, complete sequence". Additionally, circularization signatures were present indicating the completeness of the plasmid. The previous findings suggested the presence of a plasmid with a length of 3770 bp.

Finally the sequence with a length of 5386 bp had as best blast hit "*Echinostoma caproni* genome assembly E_caproni_Egypt, scaffold ECPE_contig0001929". Several blast hit results with a similar bit-score indicated the presence of a phage. This may explain the presence of circularization signatures at the ends of the sequence.

Enterobacter cloacae ECR091

PlasmidSPAdes and Recycler identified the same isolated component formed by a single contig with a length of 4.6 kbp. Best blast hit corresponded "*Salmonella enterica* subsp. enterica serovar Typhimurium str. U288 plasmid pSTU288-3, complete sequence" with the same length. Contig annotation spotted the presence of a mobilization protein (MbeC) and circularization signatures confirmed the completeness of the plasmid. A k-mer coverage ratio of 11.8 suggests the presence of a novel plasmid with a length of 4667 bp not present in the reference assembly.

Additionally, PlasmidSPAdes identified another isolated component with a contig length of 2572 bp. Best blast hit corresponded to "*Enterobacter agglomerans* ColE1-like plasmid RNA one modulator (rom) gene, complete cds" with a length of 2.49 kbp. A k-mer coverage ratio of 22.0 and the presence of circularization signatures suggests the identification of a novel plasmid.

Enterobacter cloacae ECNIH3

PlasmidSPAdes and Recycler identified the same component with a contig length of 2.49 kbp. This component is the same identified by PlasmidSPAdes in the isolate *E. cloacae* ECR091. In this case, the k-mer coverage ratio is even higher 30.9 and Recycler reported the correct size of the plasmid (2495 bp).

Klebsiella oxytoca KONIH1

PlasmidSPAdes identified an isolated component with a contig length of 3.71 kbp. Best blast hit corresponded to "*Enterobacter asburiae* strain CAV1043 plasmid pCAV1043-10, complete sequence" with a length of 10.40 kbp. The presence of circularization signatures and a high k-mer coverage ratio suggests the presence of a novel plasmid not present in the reference assembly.

Klebsiella pneumoniae strain KPNIH39

PlasmidSPAdes and Recycler identified the same sequence with a length of 5521 bp. Best blast hit corresponded to "*Enterobacter cloacae* plasmid pNE1280, complete sequence". Presence of circularization signatures and a k-mer coverage ratio of 9.1 indicated the presence of a small cryptic plasmid with a length of 5521 bp.

Table S3: Novel sequences not present in the reference genome predicted by PlasmidSPAdes and Recycler.

	pSPAdes	Recycler	cBar	k-mer coverage ratio	Blast hit	Annotation	Circularity
B. subtilis BEST195	5513	5386	Plasmid	10.3	Plasmid (CP003995)	-	✓
K. pneumoniae KPN223	4294	4167	Plasmid	0.9	Plasmid (EU932690)	-	✓
	4141	4014	Plasmid	1.5	Non significant	Mob. protein MobA	✓
	-	3478	Plasmid	1.3	Plasmid(NZ CP012489)	-	✓
E. coli JJ1886	11105	-	Chromosome	0.2	Chromosome (CP013218)	-	X
	-	2361	Plasmid	1.0	Plasmid (CP014694)	-	✓
	-	2216	Plasmid	0.8	Plasmid (Y16944)	-	✓
	1689	1634	Plasmid	0.2	Plasmid (JQ312422)	-	✓
A. veronii AVNIH1	7241	7114	Plasmid	6.3	Plasmid (KT781681)	Antitoxin RelB	✓
	1863	1736	Chromosome	15.7	Plasmid (LN853312)	-	✓
K. pneumoniae AATZP	4294	4167	Plasmid	2.4	Plasmid (CP003995)	-	✓
K. pneumoniae CAV1392	2572	2495	Plasmid	0.1	Plasmid (NC 015515)	-	✓
C. freundii CFNIH1	5487	5410	Plasmid	14.1	Plasmid (NZ_CP011613)	Relaxase MbeA	✓
E. faecium ATCC 700221	12589	12462	Plasmid	2.7	Plasmid (AB158402)	-	✓
	5513	5386	Plasmid	26.6	Phage (CP004084)	-	✓
K. pneumoniae KPN555	4175	4048	Plasmid	0.4	Plasmid (JX238446)	Relaxase MbeA	✓
	3605	3478	Plasmid	0.9	Plasmid (CP000652)	Antitoxin MazE	✓
	3001	2874	Plasmid	1.7	Plasmid (HG796369)	Plasmid recombination enzyme	✓
	2925	2798	Plasmid	2.0	Plasmid (HG796369)	-	✓
K. pneumoniae PMK1	5695	5640	Plasmid	26.0	Plasmid (LN854314)	Antitoxin IgA-2, Mob. protein MbeC	✓
	5441	5386	Plasmid	2.0	Scaffold (LL266921)	-	✓
	3825	3770	Plasmid	35.0	Plasmid (NC 019077)	-	✓
E. cloacae ECR091	4744	4667	Plasmid	11.8	Plasmid (CP004060)	Mob. protein MbeC	✓
	2572	-	Plasmid	22.0	Plasmid (AF014880)	-	✓
E. cloacae ECNIH3	2572	2495	Plasmid	30.9	Plasmid (AF014880)	-	✓
K. oxytoca KONIH1	3713	-	Chromosome	40.7	Plasmid (CP011586)	-	✓
K. pneumoniae KPNIH39	5550	5521	Plasmid	9.1	Plasmid (NC 019346)	-	✓

References

[1] Bankevich A, Nurk S, Antipov D, Gurevich Aa, Dvorkin M, Kulikov AS, et al. SPAdes: A New Genome Assembly Algorithm and Its Applications to Single-Cell Sequencing. Journal of Computational Biology. 2012;19(5):455–477.

[2] Altschul SF, Gish W, Miller W, Myers EW, Lipman DJ. Basic local alignment search tool. Journal of molecular biology. 1990 oct;215(3):403–10. Available from: http://linkinghub.elsevier.com/retrieve/pii/S0022283605803602http://www.ncbi.nlm.nih.gov/pubmed/2231712.

[3] Carattoli A, Zankari E, Garciá-Fernández A, Larsen MV, Lund O, Villa L, et al. In Silico detection and typing of plasmids using plasmidfinder and plasmid multilocus sequence typing. Antimicrobial Agents and Chemotherapy. 2014;58(7):3895–3903.

[4] Li H, Durbin R. Fast and accurate short read alignment with Burrows-Wheeler transform. Bioinformatics. 2009;25(14):1754–1760.

[5] Li H, Handsaker B, Wysoker A, Fennell T, Ruan J, Homer N, et al. The Sequence Alignment/Map format and SAMtools. Bioinformatics. 2009;25(16):2078–2079.

[6] Gurevich A, Saveliev V, Vyahhi N, Tesler G. QUAST: Quality assessment tool for genome assemblies. Bioinformatics. 2013;29(8):1072–1075.

[7] Mikheenko A, Valin G, Prjibelski A, Saveliev V, Gurevich A. Icarus: visualizer for de novo assembly evaluation. Bioinformatics. 2016;Available from: http://www.ncbi.nlm.nih.gov/pubmed/27378299.

[8] Wick RR, Schultz MB, Zobel J, Holt KE. Bandage: Interactive visualization of de novo genome assemblies. Bioinformatics. 2015;31(20):3350–3352.

[9] Rozov R, Brown Kav A, Bogumil D, Shterzer N, Halperin E, Mizrahi I, et al. Recycler: an algorithm for detecting plasmids from <i>de novo</i> assembly graphs. Bioinformatics. 2016 dec;p. btw651. Available from: https://academic.oup.com/bioinformatics/article-lookup/doi/10.1093/bioinformatics/btw651.

[10] Antipov D, Hartwick N, Shen M, Raiko M, Pevzner PA. plasmidSPAdes : Assembling Plasmids from Whole Genome Sequencing Data. Bioinformatics. 2016;.

[11] San Millan A, Heilbron K, MacLean RC. Positive epistasis between co-infecting plasmids promotes plasmid survival in bacterial populations. The ISME journal. 2014;8(3):601–12. Available from: http://dx.doi.org/10.1038/ismej.2013.182http://www.pubmedcentral.nih.gov/articlerender.fcgi?artid=3930321{&}tool=pmcentrez{&}rendertype=abstract.

[12] Arndt D, Grant JR, Marcu A, Sajed T, Pon A, Liang Y, et al. PHASTER: a better, faster version of the PHAST phage search tool. Nucleic Acids Research. 2016 jul;44(W1):W16–W21. Available from: http://www.ncbi.nlm.nih.gov/pubmed/27141966http://www.pubmedcentral.nih.gov/articlerender.fcgi?artid=PMC4987931https://academic.oup.com/nar/article-lookup/doi/10.1093/nar/gkw387.

[13] Seemann T. Prokka: Rapid prokaryotic genome annotation. Bioinformatics. 2014;30(14):2068–2069.

[14] Krumsiek J, Arnold R, Rattei T. Gepard: A rapid and sensitive tool for creating dotplots on genome scale. Bioinformatics. 2007;23(8):1026–1028.
